# Editorial and Miscellaneous

**Published:** 1861-03

**Authors:** 


### EDITORIAL.

*Atropine.*—Dr. Max Maresch, Physician to an Insane Asylum in Vienna, reports a number of cases of epilepsy treated by this powerful medicine. He states that the results of observations both by himself and others are such as to render this remedy worthy of serious attention. He recommends five drop doses of a solution containing one grain in a hundred drops of rectified alcohol. Given fasting, and from the subsequent meal coffee, tea and chocolate to be excluded as being incompatible. Treatment from sixty to ninety days; then an interruption of thirty or forty days, then continue as before.

*Fluid Extracts.*—When properly prepared, this class of remedial preparations prove very convenient and efficacious; but they are so often slovenly and carelessly separated, or entirely substituted, that much caution is requisite in their use. Intelligent druggists have assured us that the published methods of preparation are evidently in many cases, simply subterfuges for the sale of mere empiric mixtures. Dr. Samuel Percy, of New York, reporting to the State Medical Society, observes: "Chemical analysis has proved in the first place, that some of them are almost wanting in medicinal powers; that others vary very much in the amount of active ingredients contained in preparations made by the same manufacturers; that the active ingredients vary in amount in that drawn from the top and that from the bottom of the bottle; that many of them contain the nutritive elements of the plant in large quantities, which must soon cause decomposition; that decomposition has taken place in a great many of them, is proved by the presence of the confervæ growing on them." Similar objections might be urged against all extracts. It is a well known fact that even opium, in years when it is most freely afforded by the plant, has the usual proportionate amount of morphia reduced to the merest trace. This even when it is prepared with the greatest caution. The pure alkaloids should be preferred rather than any, except a *proved* extract.

---

*Poisonous Candy.*—Two cases, in one family, have recently come under our observation of quite severe symptoms of irritant metallic poisoning, from eating the richly colored candies offered for sale in the shops. The yellow colored candy (probably from the presence of orpiment) appears to have been the active agent. Cannot measures be taken to prevent this dangerous practice? It is well known that children are liable to similar attacks from sucking off the paint from their colored toys.

---

*Tallman's Adhesive Plaster.*—Our subscribers will find this article, (see third page of the cover,) a very convenient one for general use. It possesses evident advantages over the form previously commonly employed.

---

*Books and Pamphlets Received.*—DIPHTHERIA—The Fiske Fund Prize Essay; by D. D. Slade, M. D. MIASMATIC FEVER; by C. A. Logan, M. D. CONSUMPTION; by Prof. Lawson. The MOVEMENT COURSE; by Chas. F. Taylor, M. D. PAINE'S INSTITUTES OF MEDICINE, REPORT OF LONGVIEW ASYLUM, O'REILLY ON THE PLACENTA, &c.

We shall endeavor to notice these works respectively in the ensuing number.

---

*Confidential.*—A long list of arrearages has accumulated upon our books. Indeed so huge that the very idea of transcribing the items, in the shape of individual bills, causes the dexter arm of the Junior Editor, albeit accustomed to constant exercise, to twinge with rheumatic anticipations. Nevertheless, the printer's pay-day comes around monthly, certain as an inexorable fate. Will our subscribers aid us by promptly responding to this covert insinuation? Enable us to abbreviate the long list, and spare us large extra expenditure for clerk hire and postage—not to speak of that expense to which our finer sensibilities are always put by either writing or presenting the individual dun. We only breathe the suggestion over the wide field of our subscription list, as the sower scatters gently wheat upon the prairie, trusting it will return in full sheaves after a *few* days.

---

*Diphtheria.*—We regret to say that in the mortality report of Chicago, to be found on another page, we are compelled to disbelieve the item with reference to Diphtheria. The regular practitioners of the city are not responsible for even a moiety of the deaths under this head. But the quacks of all shades find this a convenient receptacle in which they can hide their fatal cases of croup, scarlatina, bronchitis, &c., &c. We repeat, Diphtheria is not epidemic, and sporadic cases are sparse and rare.

---

*Time.*—Every effort is being used to bring the *Journal* "up to time," but, practically, this has proved more difficult than supposed, when we somewhat rashly promised a speedy accomplishment of this desirable object. The prospect is now good for the next or ensuing month.

---

*War.*—Prof. McDowell, in his Valedictory at St. Louis, a few days since, proposed to go South and join the seceders' army as a surgeon. We trust he will have no occasion for carving his brethren of the *National* Med. Association.

---

—Through the kindness and liberality of Dr. A. McFarland, Supt. of the State Insane Asylum, we are enabled to mail with the present number a copy of the valuable Annual Report of that Institution. It contains matter of permanent interest.

*Popliteal Aneurism.*—Our exchanges chronicle quite a number of cases of popliteal and other aneurisms radically cured by compression. Prof. Blackman records a recent case where digital compression proved strikingly efficient. It appears to be unnecessary that pressure should be employed more than a few hours daily. The consolidated tumor left behind gradually shrivels away after the disease is cured. The results are very encouraging.

### ILLINOIS STATE MEDICAL SOCIETY.

The subscriber respectfully requests the members of the Society and the profession at large, to assist in accomplishing the object for which the Special Committee on Diseases of the Eye was appointed.

Reports of Ophthalmic cases and papers upon any subject in this department of medical science are solicited.

The Committee particularly requests papers upon the peculiar causes of conjunctival inflammation in different sections of Illinois and the adjoining States; as also the history of cases of sympathetic ophthalmitis in one eye following injuries, and chronic inflammation of the iris and choroid of the other eye.

It is hoped the profession generally will take interest in this important subject and forward all communications to the Committee, before May 1, 1861.

E. L. HOLMES, Chicago.
**P. O. Box 2175.**

COMMUNICATIONS.—All communications for insertion in the Journal, or on business matters connected therewith, may hereafter be directed to the Junior Editor,

DR. J. ADAMS ALLEN,
Box 4458, Chicago, Illinois.

### GRADUATES IN RUSH MEDICAL COLLEGE.

### SESSION 1860-61.

| STATES. | | THESES. |
|---|---|---|
| Illinois | Chas. Bunce | Hydrophobia. |
| Wisconsin | Allen S. Barndt | Intermittent Fever. |
| Wisconsin | Wm. C. Brown | Menstruation. |
| Illinois | Sidney S. Buck | Tubercles. |
| Illinois | Benj. H. Bradshaw | Typhoid Fever. |
| Connecticut | Henry S. Blood | Advancement of Med. Science. |
| Illinois | E. A. Clark | Inflammation. |
| Iowa | D. M. Cool | Repair of Fractures. |
| Nebraska | Thos. J. Dunn | Hysteria. |
| Indiana | Edward C. De Forest | Diphtheria. |
| Illinois | M. M. Eaton | ——— |
| Indiana | Geo. Egbert | Dyspepsia. |
| Indiana | Wm. B. Graham | Scarlatina. |
| Ohio | Henry J. Herrick | Insanity. |
| Maine | Zenas P. Hanson | Mental Influence. |
| Illinois | Clinton D. Henton | Blisters. |
| Iowa | Ezekeil Keith | Diphtheria. |
| Michigan | Jno. T. Keables | Tuberculosis of Liver. |
| Indiana | Enoch W. Keegan | Continued Fever. |
| Indiana | Abner D. Kimball | Hæmoptysis. |
| Iowa | Robert M. Lackey | Local Hysteria. |
| Illinois | Z. James McMaster | Water. |
| Illinois | James M. Mayfield | Science of Medicine. |
| Iowa | Henry H. Maynard | Pernicious Fever. |
| Illinois | Richard E. McVey | Circulation of Blood. |
| Illinois | John Murphy | Natural Labor. |
| Indiana | Samuel C. Owen | Placenta Prævia. |
| Illinois | Allen M. Pierce | Opium. |
| Indiana | Henry V. Passage | Pneumonia. |
| Illinois | Madison Reece | Strychnia. |
| Wisconsin | E. Fred. Russell | Blood. |
| Indiana | Edward P. Talbott | Acute Dysentery. |
| Illinois | Chas. B. Tompkins | Arsenic. |
| Illinois | Theodore W. Stull | Responsibilities of Medical Students. |
| Indiana | Israel B. Washburn | Osteology. |
| Indiana | O. G. Walker | Pneumonia. |

### HONORARY DEGREES.

Robert C. Hamill, Chicago; Theodore Hoffman, Niles.

### MORTALITY OF CHICAGO FOR JANUARY, 1861.

| DISEASE. | NO. | DISEASE. | NO. |
|---|---|---|---|
| Brain, Inflammation of... | 1 | Dysentery ............. | 1 |
| " Congestion of..... | 3 | Parturition ............. | 1 |
| Convulsions ............ | 10 | Metritis................ | 1 |
| Hydrocephalus ......... | 1 | Puerperal Fever......... | 1 |
| Delirium Tremens....... | 1 | Dropsy ................ | 7 |
| Teething.............. | 17 | Old Age................ | 3 |
| Mumps ................ | 1 | Burns.................. | 3 |
| Diphtheria ............ | 27 | Intemperance.......... | 1 |
| Croup ................ | 15 | Typhoid Fever.......... | 5 |
| Asthma................ | 1 | Scarlatina ............. | 2 |
| Pneumonia............ | 9 | Erysipelas.............. | 1 |
| Consumption .......... | 35 | Still Born.............. | 3 |
| Apoplexy, Pulmonary.... | 1 | Violence ............... | 3 |
| Cardiac Disease......... | 3 | Unknown............... | 10 |
| Enteritis .............. | 2 | | —— |
| Cholera, Infant......... | 5 | | 174 |

| AGES. | NO. |
|---|---|
| Under 1 year........... | 29 |
| Between 1 and 5 years.. | 5 |
| " 5 " 10 " .. | 12 |
| " 10 " 20 " .. | 8 |
| " 20 " 30 " .. | 8 |
| " 30 " 40 " .. | 25 |
| " 40 " 50 " .. | 18 |
| " 50 " 60 " .. | 6 |
| " 60 " 70 " .. | 3 |
| " 70 " 80 " .. | 2 |
| Unknown .............. | 5 |
| Still Born.............. | 3 |
| | —— |
| | 174 |

*Croup.*

| AGES. | NO. |
|---|---|
| Under 1 year........... | 3 |
| Between 1 and 5 years... | 10 |
| " 5 " 10 " ... | 2 |
| | —— |
| | 15 |

*Consumption.*

| AGES. | NO. |
|---|---|
| Betw. 1 and 5 years.... | 2 |
| " 5 " 10 " .... | 1 |
| " 10 " 20 " .... | 2 |
| " 20 " 30 " .... | 4 |
| " 30 " 40 " .... | 16 |
| " 40 " 50 " .... | 10 |
| | —— |
| | 35 |

*Diphtheria.*

| AGES. | NO. |
|---|---|
| Betw. 1 and 5 years.... | 19 |
| " 5 " 10 " .... | 6 |
| " 20 " 30 " .... | 1 |
| Unknown ............. | 1 |
| | —— |
| | 27 |

*Deaths in the different Divisions of the City, divided as follows:*

| | | | |
|---|---|---|---|
| North Division......... | 54 | West Division.......... | 59 |
| South " ......... | 53 | Not stated.............. | 7 |

